# Effects of Sodium Butyrate Forms on Carcass Performance, Pulmonary Hypertension, Metabolic Health and Oxidative‐Inflammatory Responses in Broiler Chickens Under Cold Stress at High Altitude

**DOI:** 10.1002/vms3.70720

**Published:** 2026-01-27

**Authors:** Behnam Ahmadipour, Sajad Pat, Fariborz Khajali, Hossein Hassanpour

**Affiliations:** ^1^ Department of Animal Sciences Faculty of Agriculture Shahrekord University Shahrekord Iran; ^2^ Department of Basic Sciences Faculty of Veterinary Medicine Shahrekord University Shahrekord Iran; ^3^ Health Equity Research Center Shahed University Tehran Iran

**Keywords:** broiler chicken, cold stress, high‐altitude, sodium butyrate

## Abstract

**Objectives:**

This study aimed to evaluate and compare the effects of non‐encapsulated sodium butyrate (SB) and encapsulated sodium butyrate (ESB) on carcass performance, pulmonary hypertension, metabolic health and oxidative‐inflammatory responses in broiler chickens exposed to cold stress at high altitude (2100 m).

**Methods:**

A total of 675 one‐day‐old male broiler chicks were randomly allocated to nine dietary treatments: a control basal diet, diets supplemented with SB (0.05% or 0.1%), ESB (0.05% or 0.1%), or their combinations. Birds were reared under a cold stress temperature regime for 42 days. Carcass traits, organ weights, the right ventricle‐to‐total ventricle weight ratio (RV:TV) and ascites mortality were assessed. Blood samples were analysed for lipid profiles, liver enzymes and oxidative stress, including malondialdehyde (MDA), nitric oxide (NO) and the heterophil‐to‐lymphocyte (H:L) ratio.

**Results:**

ESB and SB‐ESB combinations significantly improved carcass performance, increasing breast and thigh yields while reducing abdominal fat. The RV:TV ratio, an indicator of pulmonary hypertension and ascites mortality were reduced in the SB0.1% and specific combination groups. Supplementation improved metabolic health, evidenced by enhanced lipid profiles (reduced cholesterol, increased HDL) and decreased liver enzyme activities (ALT, AST, ALP). Oxidative stress was mitigated, with reduced MDA and increased NO levels across treated groups. Immunomodulatory benefits were observed, including a decreased H:L ratio.

**Conclusions:**

Dietary supplementation, particularly with ESB at 0.1%, effectively mitigated the adverse effects of high‐altitude cold stress by improving carcass yield, reducing pulmonary hypertension, enhancing metabolic and liver health and strengthening antioxidant and anti‐inflammatory responses. ESB is identified as an optimal nutritional strategy for supporting broiler production in challenging environments.

## Graphical abstract

Encapsulated sodium butyrate outperforms its non‐encapsulated form by enhancing gut health, reducing oxidative stress and pulmonary hypertension and improving carcass yield in broilers exposed to high‐altitude cold stress, offering an effective nutritional strategy for challenging environments.

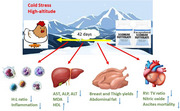

## 1 Introduction

Broiler chickens reared at high altitudes face several environmental challenges that significantly compromise their health and productivity. Among these, cold stress emerges as a critical factor, as the combination of low ambient temperatures and hypoxia (due to reduced oxygen availability at elevation) imposes substantial physiological strain (Hossain and Akter 2022). Cold stress triggers a cascade of detrimental effects, beginning with increased metabolic demand as birds expend more energy to maintain body temperature, thereby diverting nutrients away from growth and muscle development. This results in impaired growth performance, characterized by reduced weight gain and poor feed conversion efficiency (Fathi, Saeedian et al. 2025). Moreover, the cardiovascular system is significantly affected, with cold stress exacerbating pulmonary hypertension syndrome (PHS, or ascites), a major cause of mortality in high‐altitude broiler production. The reduced oxygen tension at altitude, combined with cold‐induced vasoconstriction, elevates pulmonary arterial pressure, leading to right ventricular hypertrophy and eventual heart failure (Miao et al. 2022). Concurrently, lipid metabolism is disrupted, manifesting in altered serum triglyceride and cholesterol levels, hepatic lipid accumulation and impaired energy utilization (Ahmadipour, Hasani et al. 2025).

At the cellular level, cold stress induces oxidative stress through the overproduction of reactive oxygen species (ROS), which overwhelm endogenous antioxidant defences (Ahmadipour, Isfahani et al. 2025). This oxidative damage, in turn, promotes systemic inflammation, as evidenced by elevated pro‐inflammatory cytokines (Fathi, Zarrinkavyani et al. 2025). The combined effects of metabolic dysregulation, oxidative tissue damage and chronic inflammation not only diminish carcass yield and meat quality but also increase susceptibility to infections, further undermining flock productivity and economic returns (Fathi, Zarrinkavyani et al. 2024).

Sodium butyrate (SB), the sodium salt of butyric acid, is a short‐chain fatty acid that plays a crucial role in poultry physiology and nutrition (Abd El‐Aziz et al. 2024). As an endogenous metabolite produced by microbial fermentation of dietary fibre in the lower gastrointestinal tract, SB exerts multiple beneficial effects that are particularly relevant under challenging rearing conditions. Its physiological importance stems from three primary mechanisms of action. First, SB serves as the preferred energy source for intestinal epithelial cells, supporting mucosal development and barrier function. It enhances gut integrity by upregulating tight junction proteins and stimulating mucus production, thereby helping to maintain intestinal homeostasis. These effects are significant in stress conditions where gut barrier function may be compromised (Melaku et al. 2021). Second, SB demonstrates significant immunomodulatory properties (Liu et al. 2022). It can downregulate pro‐inflammatory pathways by inhibiting nuclear factor‐kappa B signalling, thereby reducing the production of inflammatory cytokines. Concurrently, it promotes the differentiation of regulatory immune cells, helping to maintain appropriate immune responses without excessive inflammation (Sarker et al. 2025). Third, SB influences metabolic regulation at multiple levels. It enhances nutrient digestion and absorption by modulating digestive enzyme activity and intestinal morphology (Ji et al. 2023). At the cellular level, SB affects energy metabolism through its role as a histone deacetylase (HDAC) inhibitor, influencing the expression of genes involved in lipid and glucose metabolism (Yang et al. 2021). This property may be particularly valuable in cold stress conditions where metabolic demands are increased. The efficacy of SB depends largely on its formulation. Non‐encapsulated SB is rapidly absorbed in the proximal gastrointestinal tract, limiting its effects in the lower gut. Encapsulated form (ESB) is designed for delayed release, ensuring more consistent delivery throughout the intestinal tract. This characteristic is crucial for optimizing its gut health‐promoting effects (Elnesr et al. 2020).

This study aims to evaluate and compare the effects of SB and ESB on broiler chickens reared under cold stress at high altitude. Specifically, we will assess their impact on (1) carcass performance, (2) pulmonary hypertension response (right ventricular hypertrophy and ascites incidence), (3) metabolic health (hepatic enzymes and lipid profile) and (4) oxidative‐inflammatory status (lipid peroxidation and pro‐inflammatory markers). By elucidating the differential efficacy of SB formulations in mitigating high‐altitude cold stress, this research seeks to identify optimal nutritional strategies for improving broiler health and productivity in challenging environments.

## 2 Materials and Methods

### 2.1 Bird Management

This research was conducted at the experimental station located at an altitude of 2100 m above sea level. The study involved 675 one‐day‐old male Cobb broiler chicks, each with an average initial weight of 41.3 ± 0.5 g. The chicks were randomly distributed into 45 floor pens, each measuring 1.5 m^2^. The experiment was conducted with equal replication. Each of the nine dietary treatments was replicated across five pens (five replicates per treatment), with each pen containing 15 birds. Every pen was equipped with a bell drinker and a feeding trough. For experimental purposes, the birds were organized into nine groups, each comprising 75 birds spread across five pens. The birds were raised following standard management practices, except for ambient temperature, which was gradually lowered in a stepwise manner to induce cold stress: 32°C ± 1°C from Day 1 to 7, then decreased to 25°C ± 1°C for Days 8 to 14, followed by 20°C ± 1°C from Days 15 to 21 and finally maintained at 15°C ± 1°C from Day 22 until Day 42 (Ahmadipour, Isfahani et al. 2025). Throughout the study, the birds had unrestricted access to feed and water. The basal diet, primarily composed of corn and soybean meal, was formulated for three growth phases: starter, grower and finisher (Table S1). Eight experimental diets were prepared by adding SB (Adisseo Co., Antony, France) in two forms. The SB, containing 80% butyric acid and 20% sodium, was supplemented at levels of 500 mg/kg (0.05%) or 1000 mg/kg (0.1%). The ESB, consisting of 24% butyric acid, 6% sodium and 70% encapsulating material, was added at the same concentrations (500 mg/kg or 1000 mg/kg). In addition, combined treatments (COMB) with both SB and ESB were administered at ratios of 0.05% + 0.05%, 0.05% + 0.1%, 0.1% + 0.05% and 0.1% + 0.1%. Table 1 indicates the summary of dietary treatment groups. To ensure consistent electrolyte balance across all diets, NaCl and NaHCO_3_ contents were adjusted accordingly for the starter, grower and finisher phases.

**TABLE 1:** Experimental dietary treatments and group allocation.

Group	Treatment code	Dietary supplementation
1	Control	Basal diet (no additive)
2	SB0.05%	Basal diet + 0.05% non‐encapsulated sodium butyrate
3	SB0.1%	Basal diet + 0.1% non‐encapsulated sodium butyrate
4	ESB0.05%	Basal diet + 0.05% encapsulated sodium butyrate
5	ESB0.1%	Basal diet + 0.1% encapsulated sodium butyrate
6	COMB‐1	Basal diet + 0.05% SB + 0.05% ESB
7	COMB‐2	Basal diet + 0.05% SB + 0.1% ESB
8	COMB‐3	Basal diet + 0.1% SB + 0.05% ESB
9	COMB‐4	Basal diet + 0.1% SB + 0.1% ESB

Abbreviations: Comb, combination; ESB, encapsulated sodium butyrate; SB, sodium butyrate.

### 2.2 Blood Sampling, Carcass Assessment and PHS Index Measurement

At 42 days, 12 birds from each group were randomly selected for blood sampling. 4 mL of blood was collected from the brachial vein and centrifuged at 2500 × g for 10 min to obtain serum. Following blood collection, euthanasia was performed using CO_2_. The serum samples were stored at −20°C until biochemical analysis.

Carcass indices were evaluated by weighing the carcass, breast and thigh yields, as well as the liver, bursa, gizzard, spleen, abdominal fat and heart. The heart ventricles were carefully dissected and weighed to determine the right‐to‐total ventricular weight ratio (RV:TV ratio), an established marker for PHS. In addition, mortality related to PHS was recorded daily during the study, with an RV:TV ratio greater than 0.25 being used as a threshold to identify cases of pulmonary hypertension (Rostami et al. 2016).

### 2.3 Oxidative Stress, Metabolic and Inflammatory Indices Assessment

Malondialdehyde (MDA), an important indicator of lipid peroxidation, was quantified in plasma samples using the TBARS assay. Plasma was first treated with trichloroacetic acid to precipitate proteins, followed by centrifugation. The resulting supernatants were incubated with thiobarbituric acid (TBA) in a boiling water bath for 10 min. After cooling, absorbance was measured at 532 nm utilizing a Corning 480 spectrophotometer. TBARS assay values were then converted to micromolar (µM). (Tohidifar et al. 2023).

Serum nitric oxide (NO) was assessed by measuring nitrite and nitrate. Nitrate was reduced to nitrite using activated cadmium, then total nitrite was measured via the Griess reaction. After deproteinizing and preparing serum samples, they were incubated with Griess reagents, and absorbance was read at 540 nm. Results were calculated from standard curves and expressed in µM (Pirali Kheirabadi et al. 2011). All used chemicals were purchased from Sigma Chemical Co. (Louis, MO).

A blood aliquot was used to prepare smears on glass slides for differential leukocyte counting. After fixation in methanol, smears were stained with May–Grunwald and Giemsa stains within 2 to 4 h. A total of 100 leukocytes, including granular heterophils and nongranular lymphocytes, were counted to calculate the heterophil to lymphocyte (H:L) ratio. Serum samples were also analysed for biochemical parameters, including total protein, albumin, glucose, cholesterol, triglycerides, high‐density lipoprotein (HDL), alanine transaminase (ALT), aspartate transaminase (AST) and alkaline phosphatase (ALP) using standard kits from Pars Azmun Co. (Karaj, Iran).

### 2.4 Statistical Analysis

The data were analysed using the General Linear Model (GLM) procedure in SAS software (2007) under a completely randomized design. The pen was considered the experimental unit for all analyses (*n* = 5 per treatment). The statistical model for data was expressed as: *Y*_ij_ = *μ* + *T*_i_ + *e*_ij_. For other variables, the model was formulated as *Y*_ijk_ = *μ* + *T*_i_ + *e*_ij_ + *ɛ*_ijk_. In these equations, *Y*_ij_ and *Y*_ijk_ represent the observations; μ denotes the overall mean; *T*_i_ indicates the treatment effect; *e*_ij_ accounts for random error and *ɛ*_ijk_ represents subsampling error. Differences between means were evaluated using Duncan's multiple‐range test (*p* < 0.05).

## 3 Results

### 3.1 Carcass Performance

Table 2 presents the effect of SB and ESB on the carcass performance parameters of broiler chickens raised at high altitudes under cold stress after 42 days. The carcass and breast yields were higher in ESB0.05%, ESB0.1%, COMB‐1, COMB‐2, COMB‐3 and COMB‐4 than in the control group (*p* < 0.05), while these parameters did not change in SB0.05% and SB0.1% groups compared to the control group (*p* > 0.05). Thigh yield increased and abdominal fat decreased (*p* < 0.05) in ESB 0.1% and all combined supplemented groups but remained unchanged in SB 0.05%, SB 0.1% and ESB 0.05% groups relative to the control (*p* > 0.05).

**TABLE 2:** Effect of sodium butyrate and encapsulated sodium butyrate on the carcass indices of broiler chickens after 42 days.

BW (%)	Control	SB 0.05%	SB 0.1%	ESB 0.05%	ESB 0.1%	COMB‐1	COMB‐2	COMB‐3	COMB‐4	SEM	*p*‐value
Carcass yield	73.7^d^	73.9^d^	74.3^cd^	74.6^bc^	75.1^ab^	75.1^ab^	75.4^a^	75.4^a^	75.4^a^	0.21	> 0.001
Breast yield	28.9^c^	29.6^bc^	29.7^abc^	30.1^ab^	30.1^ab^	30.4^a^	30.5^a^	30.1^ab^	30.2^ab^	0.26	0.006
Thigh yield	27.2^b^	27.6^ab^	27.5^ab^	27.6^ab^	28.1^a^	28.1^a^	28.1^a^	28.1^a^	27.9^a^	0.19	0.025
Liver	2.20^a^	2.11^bc^	2.04^bcd^	2.09^bc^	2.01^cd^	1.88^e^	1.93^de^	2.09^bc^	2.15^b^	0.037	> 0.001
Spleen	0.096^e^	0.104^de^	0.118^abc^	0.115^bc^	0.125^ab^	0.123^ab^	0.130^a^	0.117^bc^	0.110^cd^	0.003	> 0.001
Bursa	0.064^c^	0.070^bc^	0.082^abc^	0.073^bc^	0.082^abc^	0.085^ab^	0.094^a^	0.099^a^	0.087^ab^	0.006	0.008
Gizzard	1.90^e^	2.11^de^	2.19^cd^	2.26^cd^	2.31^bcd^	2.35^abcd^	2.43^abc^	2.55^ab^	2.60^a^	0.080	0.001
Abdominal fat	1.22^a^	1.11^ab^	1.16^ab^	1.08^ab^	0.94^bc^	0.94^bc^	0.94^bc^	0.84^c^	0.72^c^	0.072	< 0.001
heart	0.74^a^	0.72^ab^	0.69^c^	0.69^c^	0.68^c^	0.67^cd^	0.65^d^	0.70^bc^	0.70^bc^	0.008	> 0.001
RV	0.134^a^	0.124^ab^	0.111^cd^	0.112^bcd^	0.108^cd^	0.104^d^	0.092^e^	0.114^bcd^	0.120^bc^	0.003	> 0.001
TV	0.486^a^	0.480^a^	0.453^b^	0.439^bc^	0.426^c^	0.424^c^	0.404^d^	0.432^c^	0.452^b^	0.005	> 0.001
RV:TV ratio	0.27^a^	0.25^ab^	0.24^bc^	0.25^ab^	0.25^abc^	0.24^bc^	0.22^c^	0.26^ab^	0.26^ab^	0.008	0.032
Ascite mortalities	26^a^	20^abc^	18^abc^	14^c^	16^bc^	16^bc^	16^bc^	18^abc^	24^ab^	2.66	0.041

*Note*: Superscript alphabets (a–e) show significant differences between groups in each row (*p* < 0.05). COMB‐1, SB 0.05% + ESB 0.05%; COMB‐2, SB 0.05% + ESB 0.1%, COMB‐3, SB 0.1% + ESB 0.05%; COMB‐4, SB 0.1% + ESB 0.1%.

Abbreviations: BW, body weight; ESB, encapsulated sodium butyrate; RV, right ventricle; RV:TV, right ventricle to total ventricle weight ratio; SB, sodium butyrate; SEM, standard error of mean; TV, total ventricle.

The liver weight was decreased in all supplemented groups, whereas spleen and gizzard weights increased in all supplemented groups except for SB 0.05% compared to the control (*p* < 0.05). Bursa weight showed a significant increase only in the combined supplementation groups, with no changes observed in the SB 0.05%, SB 0.1%, ESB 0.05% and ESB 0.1% groups (*p* < 0.05). The heart, right ventricle and left ventricle weights decreased in all supplemented groups except for SB0.05% compared to the control (*p* < 0.05). The RV:TV ratio was only lower in SB0.1%, COMB‐1 and COMB‐2 groups than in the control group (*p* < 0.05). It did not change in SB0.05%, ESB0.05%, ESB0.1%, COMB‐3 and COMB‐4 groups compared to the control group (*p* > 0.05).

The ascites mortality was only lower in ESB0.05%, ESB0.1%, COMB‐1 and COMB‐2 groups than in the control group (*p* < 0.05). It did not change in SB0.05%, SB0.1%, COMB‐3 and COMB‐4 groups compared to the control group (*p *> 0.05).

### 3.2 Blood Biomarkers

Table 3 indicates the effect of SB and encapsulated sodium butyrate on different biomarkers of broiler chickens after 42 days.

**TABLE 3:** Effect of sodium butyrate and encapsulated sodium butyrate on the biomarkers of broiler chickens after 42 days.

Metabolites	Control	SB 0.05%	SB 0.1%	ESB 0.05%	ESB 0.1%	COMB‐1	COMB‐2	COMB‐3	COMB‐4	SEM	*p*‐value
Cholesterol (mg/dL)	127^a^	124^a^	125^a^	126^a^	125^a^	124^a^	119^ab^	112^b^	118^ab^	3.08	0.027
Triglycerides (mg/dL)	75.6^bcd^	72.6^d^	74.0^cd^	76.0^bc^	77.8^ab^	76.2^bc^	80.0^a^	78.6^ab^	80.8^a^	1.03	> 0.001
HDL (mg/dL)	72.7^c^	74.8^bc^	78.8^abc^	79.4^ab^	79.1^ab^	78.6^abc^	82.0^a^	82.2^a^	84.0^a^	2.01	0.007
Malondialdehyde (µM)	3.27^a^	3.81^ab^	2.52^abc^	2.38^bc^	2.32^bc^	1.70^c^	1.69^c^	1.76^c^	1.75^c^	0.266	> 0.001
Nitric oxide (µM)	1.82^c^	3.19^b^	3.68^ab^	4.04^ab^	4.31^a^	4.05^ab^	4.29^a^	4.33^a^	4.21^a^	0.309	> 0.001
Haematocrit (%)	44.2^a^	42.4^ab^	41.8^ab^	40.4^b^	40.4^b^	40.0^b^	40.4^b^	41.8^ab^	41.8^ab^	0.808	0.018
Total protein g/dL	3.82^d^	3.94^dc^	3.96^dc^	4.02^bc^	3.96^dc^	4.00^dc^	4.24^a^	4.14^ab^	4.14^ab^	0.048	> 0.001
Albumin g/dL	2.30^c^	2.34^bc^	2.36^bc^	2.36^bc^	2.38^abc^	2.38^abc^	2.44^ab^	2.50^a^	2.48^ab^	0.038	0.015
ALT (IU/L)	3.40^a^	3.40^a^	3.00^b^	2.60^c^	3.00^b^	2.60^c^	2.60^c^	3.40^a^	3.20^ab^	0.117	> 0.001
AST (U/L)	280^a^	245^cd^	243^cd^	252^bc^	238^cd^	231^d^	233^d^	254^bc^	265^ab^	5.49	> 0.001
ALP (U/L)	1184^a^	1022^ab^	926^bc^	884^bc^	871^bc^	861^bc^	781^c^	801^c^	944^bc^	58.2	> 0.001
Glucose (mg/dL)	240^c^	246^c^	252^bc^	268^ab^	268^ab^	265^ab^	268^ab^	272^a^	274^a^	5.57	> 0.001
Heterophil (%)	33.6^a^	30.8^ab^	29.8^abc^	28.4^abc^	26.4^bc^	27.2^bc^	25.2^c^	30.4^abc^	29.2^abc^	1.69	0.044
Lymphocyte (%)	61.2^c^	63.6^bc^	68.6^ab^	70.4^a^	70.8^a^	70.0^ab^	71.6^a^	66.8^abc^	68.0^ab^	2.08	0.016
H:L ratio	0.57^a^	0.48^ab^	0.43^b^	0.40^b^	0.37^b^	0.38^b^	0.35^b^	0.45^ab^	0.43^b^	0.04	0.034

*Note*: Superscript alphabets (a–e) shows significant differences between groups in each row (*p* < 0.05). COMB‐1, SB 0.05% + ESB 0.05%; COMB‐2, SB 0.05% + ESB 0.1%, COMB‐3, SB 0.1% + ESB 0.05%; COMB‐4, SB 0.1% + ESB 0.1%.

Abbreviations: ALP, alkaline phosphatase; ALT, alanine transaminase; AST, aspartate transferase; ESB, encapsulated sodium butyrate; H:L, heterophil to lymphocyte; HDL, high‐density lipoprotein; RV, right ventricle; RV:TV, Right ventricle to total ventricle weight ratio; SB, sodium butyrate; SEM, standard error of mean; TV, total ventricle.

Among lipid and metabolic biomarkers, the cholesterol only decreased in the COMB‐3 group; triglycerides only increased in the COMB‐2 and COMB‐4 groups; HDL only increased in ESB0.05%, ESB0.1%, COMB‐2, COMB‐3 and COMB‐4 groups; the haematocrit values only decreased in ESB0.05%, ESB0.1%, COMB‐1 and COMB‐2 groups; total protein levels only increased in ESB0.05%, COMB‐2, COMB‐3 and COMB‐4 groups; albumin levels only increased in COMB‐2, COMB‐3 and SB0.1% + ESB0.1%; the glucose only increased ESB0.05%, ESB0.1% and combined SB and ESB groups compared to the control group (*p* < 0.05).

Among liver enzymes, the ALT activity only decreased in SB0.1%, ESB0.05%, ESB0.1%, COMB‐1 and COMB‐2; the AST activity decreased in all supplemented groups except for the COMB‐4 group; the ALP activity decreased in all supplemented groups except for the SB0.05% group compared to the control group (*p* < 0.05).

Among inflammatory parameters, the heterophils only decreased in the ESB0.1%, COMB‐1, COMB‐2 groups; the lymphocytes increased and the H:L ratio decreased in all supplemented groups except for the SB0.05% and COMB‐3 groups compared to the control group (*p* < 0.05).

The MDA levels only decreased in ESB0.05%, ESB0.1% and combined SB and ESB groups compared to the control group, while NO levels increased in all supplemented groups (*p* < 0.05).

## 4 Discussion

This study revealed distinct formulation‐dependent effects of SB on broiler performance under high‐altitude cold stress. The superior performance of ESB formulations and SB‐ESB combinations over non‐encapsulated SB can be attributed to fundamental differences in their bioavailability and site of action within the gastrointestinal tract (Antongiovanni et al. 2007). While non‐encapsulated SB is rapidly absorbed in the proximal gut (e.g., crop and gizzard), its beneficial effects are limited to these upper regions. In contrast, the encapsulation technology confers stability, protecting the core butyrate from premature absorption and metabolism in the acidic environment of the upper digestive tract. This ensures delayed release and targeted delivery of active butyrate to the distal small intestine and cecum (Liu et al. 2021). The distal gut is a critical site for nutrient absorption, immune modulation and the fermentation that produces other beneficial short‐chain fatty acids. By consistently supplying butyrate to these regions, ESB more effectively supports the integrity of the intestinal mucosal barrier, upregulates tight junction proteins and promotes a healthy gut microbiome. A robust gut barrier is particularly crucial under cold stress (Zhou et al. 2021), which can compromise intestinal integrity and lead to systemic inflammation. Furthermore, the sustained release from ESB provides a more stable and prolonged metabolic stimulus, optimizing energy harvest and nutrient partitioning towards growth rather than fat deposition, as evidenced by the higher breast yield and lower abdominal fat. Therefore, the enhanced efficacy of ESB is not merely a function of dosage but of optimal pharmacokinetics, ensuring that the right amount of butyrate reaches the right locations at the right time to exert its full spectrum of benefits (El‐Saadony et al. 2022).

Changes in organ weights demonstrated distinct responses to butyrate formulations, with liver weight decreasing in all supplemented groups, reflecting butyrate's role in enhancing hepatic β‐oxidation and reducing lipid accumulation. Correspondingly, the reduction in cholesterol levels observed in the SB0.1% + ESB0.05% group and the increase in HDL in the ESB and combined SB‐ESB groups indicate that butyrate influences lipid metabolism by promoting β‐oxidation and inhibiting lipogenesis. These findings align with previous research showing butyrate's positive effects on lipid profiles (Coppola et al. 2021). However, the observed increase in triglycerides in certain SB‐ESB groups (COMB‐2 and COMB‐4) may be explained by an adaptive metabolic response to cold stress. Under cold‐induced thermogenesis, the liver increases the synthesis of very‐low‐density lipoproteins (VLDL) to transport energy‐rich lipids from the liver to peripheral tissues for heat production (Odhiambo 2004). We hypothesize that the specific combination of SB and ESB in these groups provided a synergistic metabolic stimulus that enhanced this process, leading to a transient rise in circulating triglycerides as part of a heightened energy‐mobilization state, without the concurrent hepatic lipid accumulation indicated by the reduced liver weights. Notably, spleen weight increased in all groups except SB0.05%, while bursa weight only increased in combination groups (SB+ESB), suggesting formulation‐dependent immunomodulation. The consistent gizzard weight elevation across treatments supports butyrate's known stimulatory effect on digestive organ development (Siddiqui and Cresci 2021). Cardiac measurements showed reduced ventricular weights in most supplemented groups, with the most pronounced RV:TV ratio improvements occurring in SB0.1% and specific combination groups (COMB‐1, COMB‐2), correlating with decreased ascites mortality. These organ‐specific responses demonstrate that SB at 0.1% concentration provided optimal cardiovascular protection while maintaining carcass yields, whereas combination treatments showed superior immune‐modulating effects but compromised breast yield. The differential organ responses likely reflect variations in butyrate's bioavailability and tissue‐specific actions under cold stress conditions, with SB showing better systemic absorption for metabolic effects and ESB combinations favouring gut‐associated immune modulation (Anshory et al. 2023).

The reduction in RV:TV ratio observed in SB0.1% and specific SB‐ESB combination groups demonstrates butyrate's protective effect against PHS (Xu et al. 2025). We propose that this cardioprotection is mediated by a multifaceted mechanism operating through the gut‐heart axis. First, as a HDAC inhibitor, butyrate can upregulate the expression of endothelial nitric oxide synthase (eNOS), enhancing the production of NO (a powerful vasodilator) (Stempelj et al. 2007). The concurrent elevation in serum NO levels across all supplemented groups strongly supports this mechanism, suggesting improved pulmonary vasodilation that directly counteracts hypoxic vasoconstriction (Khajali 2022). Second, butyrate's potent anti‐inflammatory properties, likely mediated via the inhibition of the NF‐κB pathway, reduce the systemic levels of pro‐inflammatory cytokines (e.g., TNF‐α, IL‐1β), which are known to contribute to endothelial dysfunction and vascular remodelling (Liu et al. 2022). Third, by improving gut barrier integrity, butyrate reduces the translocation of gut‐derived endotoxins into the portal circulation (Siddiqui and Cresci 2021), thereby mitigating the chronic low‐grade inflammation that exacerbates pulmonary hypertension. Finally, butyrate serves as a primary energy substrate for colonocytes and enhances mitochondrial function, which may improve overall metabolic efficiency and reduce oxidative stress as a key driver of pulmonary arterial cell proliferation. The superior performance of ESB in this regard underscores the importance of sustained butyrate delivery to the lower gut for optimal modulation of this gut‐heart axis. This integrated mechanism, combining vasodilation, anti‐inflammatory action and enhanced metabolic health, provides a compelling explanation for the efficacy of butyrate in alleviating PHS (Du et al. 2021).

The significant reduction in liver enzymes (ALT, AST and ALP) across supplemented groups indicates enhanced hepatic protection, with the concurrent decrease in H:L ratio suggesting a systemic attenuation of stress responses. This hepatoprotective effect likely operates through a dual mechanism: (1) butyrate's direct antioxidant capacity, evidenced by reduced MDA levels, preserves hepatocyte integrity against oxidative insults, while (2) its immunomodulatory properties, reflected in the improved H:L ratio, mitigate inflammation‐induced liver damage (Amiri et al. 2022; Liao et al. 2021). The powerful effects in ESB and SB‐ESB groups imply that sustained butyrate release may simultaneously optimize hepatic antioxidant defences (reducing enzyme leakage) and rebalance leukocyte populations, creating a more favourable systemic environment for liver function under cold stress conditions. This interconnected improvement in both hepatic biomarkers and H:L ratio underscores butyrate's capacity to address multiple physiological consequences of high‐altitude rearing through integrated antioxidant and anti‐stress pathways, echoing the effects of other functional supplements like black cumin seed (Fathi et al. 2024b) and guanidinoacetic acid (Fathi, Saeedyan et al. 2024).

## 5 Conclusion

This study demonstrates that SB supplementation, particularly in its ESB, effectively counteracts the negative impacts of cold stress and high‐altitude conditions in broiler chickens. The key findings show that ESB and combined SB‐ESB treatments significantly enhanced carcass performance by increasing breast and thigh yields while reducing abdominal fat. These formulations also provided superior cardiovascular protection, as indicated by reduced pulmonary hypertension (RV:TV ratio) and lower ascites mortality, likely through improved pulmonary vasodilation and antioxidant effects. Metabolically, butyrate supplementation improved lipid profiles and liver function while reducing oxidative stress markers. The immunomodulatory benefits were evident through reduced inflammation and improved stress responses. Notably, ESB at 0.1% concentration showed the most consistent benefits, suggesting it as an optimal formulation for high‐altitude poultry production. These findings highlight the potential of ESB as a nutritional intervention to enhance broiler health and productivity under environmental stress. Future research should explore long‐term effects and economic viability to facilitate practical implementation in commercial poultry operations.

## Author Contributions

**Behnam Ahmadipour**: conceptualization, data curation, formal analysis, investigation, methodology, supervision, writing manuscript. **Sajad Pat**: data curation, methodology. **Fariborz Khajali**: conceptualization, formal analysis, investigation. **Hossein Hassanpour**: conceptualization, writing manuscript.

## Funding

The authors have nothing to report.

## Ethics Statement

The study underwent an ethical review and was approved (approved code: IR.SKU.23212) by the local Ethics Committee of Shahrekord University, and was carried out at the experimental facility of this university, situated at an altitude of 2100 m above sea level in Shahrekord, Iran.

## Conflicts of Interest

The authors declare no conflicts of interest.

## Supporting information




**Table S1**: Composition of the basal diet fed to broilers (Ross 308) from 1 to 42 days of age.

## Data Availability

Data are available upon request.
