# Supplementary material for: Effects of Sodium Butyrate Forms on Carcass Performance, Pulmonary Hypertension, Metabolic Health and Oxidative‐Inflammatory Responses in Broiler Chickens Under Cold Stress at High Altitude
Source: Vet Med Sci. 2026 Jan 27;12(1):e70720. doi: 10.1002/vms3.70720 (PMC12840551; doi:10.1002/vms3.70720)
Supplement: Supplementary file 1 — Table S1: Composition of the basal diet fed to broilers (Ross 308) from 1 to 42 days of age. [file VMS3-12-e70720-s001.docx]

**Table S1.**  Composition of the basal diet fed to broilers (Ross 308) from 1 to 42 days of age

| Item (% unless noted) | | Starter (1–10d) | Grower (11-22d) | Finisher (23–42d) |
| --- | --- | --- | --- | --- |
| Corn | | 53.53 | 57.53 | 63.34 |
| Soybean meal (44% CP) | | 370.5 | 35.8 | 295 |
| Corn Gluten Meal | | 3.46 | - | - |
| Soy oil | | 1.4 | 2.60 | 3.4 |
| Dicalcium phosphate | | 2.15 | 1.91 | 1.65 |
| CaCO3 | | 0.68 | 0.61 | 0.57 |
| Salt | | 0.16 | 0.20 | 0.2 |
| Na-Bicarbonate | | 0.29 | 0.24 | 0.25 |
| DL-Methionine | | 0.33 | 0.295 | 0.27 |
| L-Lysine | | 0.28 | 0.18 | 0.18 |
| L-Threonine | | 0.11 | 0.1 | 0.09 |
| Choline Chloride | | 0.06 | 0.04 | 0.05 |
| Mineral supplement**^⁕^** | | 0.25 | 0.25 | 0.25 |
| Vitamin supplement **^⁕⁕^** | | 0.25 | 0.25 | 0.25 |
| Calculated composition | |  |  |  |
| Dry Matter | | 89.026 | 88.934 | 88.920 |
| AME (kcal/kg) | | 2880.00 | 2954.00 | 3072.0 |
| CP | | 22.85 | 20.48 | 18.16 |
| Met | | 0.651 | 0.574 | 0.528 |
| Met+Cys | | 0.930 | 0.833 | 0.764 |
| Lys | | 1.23 | 1.110 | 0.980 |
| Thr | | 0.824 | 0.744 | 0.657 |
| Arg | | 1.33 | 1.252 | 1.087 |
| Na | | 0.157 | 0.157 | 0.157 |
| Cl | | 0.204 | 0.204 | 0.204 |
| K | | 0.931 | 0.926 | 0.817 |
| Na+K–Cl (mEq/kg) | | 245.10 | 247.82 | 239.92 |
|  | **^⁕^** Provided the following per kg of diet: vitamin A (trans retinyl acetate), 3600IU; vitamin D3 (cholecalciferol), 800 IU; vitamin E (dl-α-tocopheryl acetate), 7.2 mg; vitamin K3, 1.6 mg; thiamine, 0.72 mg; riboflavin, 3.3 mg; niacin, 0.4 mg; pyridoxin, 1.2 mg; cobalamine, 0.6 mg; folicacid, 0.5 mg; choline chloride, 200 mg.  **^⁕⁕^** Provided the following per kg of diet: Mn (from MnSO4-H2O), 40mg; Zn (from ZnO), 40mg; Fe (from FeSO4-7H2O), 20mg; Cu (from CuSO4-5H2O), 4 mg; I [from Ca (IO3)2-H2O], 0.64 mg; Se (from sodium selenite),0.08 mg. | | | |
